# Latent Profiles of Online Gambling and Sports Betting Participation and Psychological Distress Among Young People with Reduced Mobility

**DOI:** 10.3390/healthcare14213807

**Published:** 2026-11-07

**Authors:** Raquel Suriá-Martínez, Fernando García-Castillo, Carmen López-Sánchez, Esther Villegas-Castrillo, José A. García del Castillo

**Affiliations:** 1 Department of Communication and Social Psychology, University of Alicante, 03690 Alicante, Spain; mc.lopez@ua.es; 2 Department of General Didactics and Specific Didactics, University of Alicante, 03690 Alicante, Spain; fgarciadelcastillo@ua.es; 3 Department of Social Work and Social Services, University of Alicante, 03690 Alicante, Spain; esther.villegas@ua.es; 4 Department of Health Psychology, University of Miguel Hernandez, 03202 Elche, Spain; jagr@umh.es

**Keywords:** online gambling, young people with reduced mobility, risk profiles, anxiety, depression, prevention

## Abstract

**Background/Objectives**: This study aimed to identify latent profiles of online gambling participation among young people with reduced mobility and examine their associations with anxiety and depression, as well as the relationship between gambling-related risk and psychological distress. **Methods**: A total of 318 young people with reduced mobility were recruited through Spanish disability-related organizations. A cross-sectional design was used. Latent profile analysis (LPA) was conducted based on participation frequency and time spent per gambling episode. Differences in anxiety and depression across profiles and associations between gambling-related risk, assessed using the Massachusetts Gambling Screen (MAGS), and psychological distress were examined. **Results**: Three latent profiles of online gambling and sports betting participation were identified among young people with reduced mobility: Infrequent and non-regular participation (47.2%), Frequent and moderate participation (39.3%), and Frequent and intensive participation (13.5%). Statistically significant differences in anxiety and depression were observed across the three profiles, with higher scores among participants characterized by more frequent and intensive participation. Furthermore, MAGS scores were positively correlated with both anxiety and depression, indicating that higher gambling-related risk was associated with greater levels of emotional difficulties. **Conclusions**: These findings highlight the importance of developing accessible prevention, early detection, and support strategies tailored to the needs of young people with reduced mobility. Participation profiles and gambling-related risk represent distinct but complementary dimensions that may help guide future research and prevention initiatives.

## 1. Introduction

Disability should be understood in terms of the interaction between health conditions and personal, social, and environmental factors that may facilitate or constrain participation in different areas of life [1]. Within this context, digital environments can promote social participation and leisure by reducing certain physical and accessibility barriers. However, they may also increase exposure to potentially harmful activities, including online gambling, such as online games of chance (e.g., roulette and blackjack) and online sports betting. Research on Internet Gaming Disorder has also highlighted the relevance of problematic online gaming to mental health among young people [2], although this behaviour is conceptually distinct from online gambling.

These activities involve wagering money on uncertain outcomes and are conceptually distinct from video games, which do not necessarily involve monetary gambling. In the present study, the term online gambling refers specifically to these forms of monetary gambling conducted through online platforms. For people with disabilities, online gambling platforms may serve recreational and social functions, while simultaneously involving potential risks associated with participation [3,4].

Online gambling has several characteristics that increase its accessibility, including continuous availability, access through mobile devices, relative anonymity, the diversity of gambling modalities, and the ease of conducting financial transactions [4,5]. These characteristics may be particularly relevant for young people with reduced mobility, for whom digital environments may provide an accessible leisure alternative. Nevertheless, participation in online gambling activities, including online games of chance and online sports betting, is not homogeneous, and different forms of participation may be characterized by distinct behavioral patterns and associated factors [5,6,7].

Research specifically examining the relationship between disability and gambling remains limited, although several studies have reported associations between functional disability and gambling-related problems [8,9]. Longitudinal research has also highlighted the importance of considering disability-related circumstances when examining gambling disorder [10], while Jang et al. [11] emphasized the need for further research into gambling-related harms among people with disabilities. In Spain, Suriá-Martínez et al. [12,13,14] have documented participation in online gambling activities, including online games of chance and online sports betting, and its association with cognitive distortions and psychopathological symptoms among students and young people with disabilities.

These findings reinforce the need to examine participation patterns specifically among young people with motor disabilities.

Gambling behavior is not a homogeneous phenomenon. The pathways model proposed by Blaszczynski and Nower [15] describes different trajectories toward problem gambling, while subsequent research using classification approaches has provided evidence of heterogeneity among gamblers [4,16,17]. Using a revised version of this model, Nower et al. [18] identified three distinct subtypes and found that one of these subtypes was characterized by higher levels of anxiety and depression and by gambling behavior associated with coping with stress. These findings support the relevance of examining different patterns of gambling participation. However, the empirical profiles identified in the present study should not be considered equivalent to the theoretical subtypes proposed by the pathways model, as they are derived exclusively from gambling frequency and time spent participating in online gambling activities. The relationship between gambling and psychological distress can also be considered from theoretical frameworks that incorporate emotional and cognitive processes. The I-PACE model proposes that addictive behaviors related to Internet use may emerge from the interaction between individual characteristics, affective states, cognitive processes, and executive control mechanisms [19,20]. In this regard, difficulties in emotion regulation have been associated with problem gambling, particularly with difficulties accepting negative emotions, controlling impulses, and accessing adaptive regulatory strategies [21,22]. Similarly, several studies have documented the co-occurrence of problem gambling with anxiety and depressive symptoms [23,24,25].

Anxiety and depression are also important psychological outcomes among young people with physical disabilities. Research involving young people with cerebral palsy and other physical conditions has identified mental health difficulties and highlighted the need to address psychological well-being specifically within this population [26,27,28]. Likewise, a systematic review of psychological interventions for adolescents with physical disabilities identified anxiety and depressive symptoms among the main psychological outcomes examined [29].

Taken together, the available evidence highlights the need to examine heterogeneity in online gambling participation, including participation in online games of chance and online sports betting, and its potential association with psychological distress among young people with reduced mobility. Accordingly, the present study pursued four objectives: (1) to describe the frequency of participation in online gambling activities, time spent per gambling episode, and levels of gambling-related risk; (2) to explore the potential existence of distinct profiles of online gambling participation intensity based on participation frequency and time spent per gambling episode; (3) to examine differences in anxiety and depression levels across the latent participation profiles; and (4) to examine the associations between online gambling risk and levels of anxiety and depression.

Based on these objectives, the following research hypotheses were formulated:

**H1.**  *Online gambling participation among young people with reduced mobility varies in terms of participation frequency and time spent per gambling episode.*

**H2.**  *Distinct latent profiles of online gambling participation intensity can be identified based on participation frequency and time spent per gambling episode.*

**H3.**  *Anxiety and depression levels are expected to differ significantly across the latent participation profiles.*

**H4.**  *Online gambling risk is expected to be positively associated with anxiety and depression levels.*

## 2. Materials and Methods

### 2.1. Participants

The sample consisted of 318 young people with reduced mobility, aged 18–35 years (*M* = 24.1, *SD* = 4.6), recruited through Spanish organizations and entities working with people with physical and motor disabilities.

The inclusion criteria were being aged 18 years or older and having a documented physical condition associated with a disability or significant mobility limitations. Participants were eligible if they provided either an official administrative certificate recognizing their degree of disability or medical documentation confirming their physical condition and associated mobility limitations. As medical documentation and official administrative recognition of disability are distinct in Spain, participants could be included even if they did not have an officially recognized degree of disability.

Regarding disability status, 55.6% of participants had an officially recognized degree of disability of 65% or higher, and 31.1% had a recognized degree ranging from 33% to 64%. The remaining 13.3% had medical documentation of their physical condition and associated mobility limitations but no officially recognized degree of disability.

Regarding sociodemographic characteristics, 53% of participants were men and 47% were women. In terms of educational attainment, 65% had completed primary education or equivalent studies (EGB/ESO), while 35% had completed secondary education.

Participation was voluntary, and all participants provided informed consent before completing the study instruments. Anonymity and data confidentiality were ensured throughout the research process. The study was conducted in accordance with the ethical principles of the Declaration of Helsinki and the provisions of the General Data Protection Regulation (EU) 2016/679.

### 2.2. Instruments

To assess the frequency of participation in online gambling and betting activities and the time spent per episode, two ad hoc items were specifically designed and included in the study. The first assessed participation frequency, with four response options: approximately once a month, several times a month, several times a week, and every day. The second assessed the duration of each gambling episode as an indicator of participation intensity, with four response options: less than 20 min, 20–39 min, 40–59 min, and 60 min or more.

Gambling-related risk was assessed using the *Massachusetts Gambling Screen* (MAGS) [30], a 19-item screening instrument designed to identify indicators associated with problematic gambling behavior. The Spanish-language version adapted by Secades et al. [31] was used in this study. For the purposes of the present research, the instrument was specifically adapted to focus on participation in online gambling and betting. The assessment referred to participants’ involvement in online gambling and betting activities over a one-month period. Based on the scores obtained, participants were classified into three risk levels according to the seven-item scoring criterion: gambling problems (scores ≥ 2), transitional status (scores ≥ 0 and <2), and no gambling problems (negative scores). The Spanish version adapted to include specific references to online gambling, which has demonstrated adequate internal consistency (α = 0.86), was used. In the present sample, the scale showed an internal consistency of α = 0.83.

Emotional problems were assessed using the *Symptom Assessment-45 Questionnaire* (SA-45) [32], in the Spanish adaptation by Sandín et al. [33]. The SA-45 is a multidimensional 45-item instrument designed to assess different psychological symptoms using a response scale ranging from 0 (*not at all*) to 4 (*very much or extremely*), referring to symptoms experienced during the previous week.

Although the instrument assesses nine dimensions—depression, anxiety, somatization, obsessive-compulsive symptoms, interpersonal sensitivity, hostility, phobic anxiety, paranoid ideation, and psychoticism—the present study specifically examined the anxiety and depression dimensions in accordance with the research objectives. The original instrument demonstrated satisfactory reliability across all subscales, with adequate internal consistency for the depression and anxiety dimensions (α = 0.84 and α = 0.90, respectively). In the present sample, the depression dimension showed an internal consistency of α = 0.82, whereas the anxiety dimension yielded α = 0.78.

### 2.3. Procedure

The study followed a quantitative, cross-sectional, non-experimental design. Before data collection commenced, the research project was reviewed and approved by the Ethics Committee of the University of Alicante. The approved procedure included providing participants with prior information about the study, ensuring that participation was voluntary, guaranteeing data confidentiality, and obtaining informed consent before administering the study instruments.

Participants were recruited through the collaboration of various Spanish organizations and entities working with people with physical and motor disabilities. The inclusion criteria were being aged 18 years or older, having a medical certificate or an official certificate issued by the Spanish Social Security system confirming the existence of a disability, and participating in online gambling activities at least once a month. Individuals who did not meet any of these criteria were excluded from the study. Potential participants were provided with information about the study, the inclusion criteria, and the data collection procedure.

The questionnaires were administered in person. When participants’ mobility or accessibility needs required it, they were offered the option of completing the questionnaires online. Before participation began, all eligible participants received information about the study objectives, the nature of their participation, and the confidentiality measures in place. Those who agreed to participate subsequently provided informed consent in accordance with the procedure approved by the Ethics Committee of the University of Alicante.

Once data collection had been completed, the data were coded, screened, and prepared for statistical analysis, while maintaining the confidentiality and anonymity of participants throughout the process.

### 2.4. Data Analysis

To address the first objective, descriptive analyses were conducted using IBM SPSS Statistics (SPSS 19.0.0.0). Absolute frequencies (*n*) and percentages (%) were calculated to describe participation frequency in online gambling activities, time spent per gambling episode, and gambling-related risk levels.

Participation frequency was categorized into four options: approximately once a month, several times a month, several times a week, and every day. Time spent per episode was classified into four categories: less than 20 min, 20–39 min, 40–59 min, and 60 min or more. The percentages for each category were calculated relative to the total sample (*N* = 318), and the results were presented in frequency distribution tables.

Gambling-related risk was assessed using the total score on the Massachusetts Gambling Screen (MAGS). Based on the established cut-off scores, participants were classified into three categories: low or no risk (MAGS score < 3), moderate risk (score 3–5), and high risk or probable pathological gambling (score ≥ 6). Absolute frequencies and percentages were calculated for each category to describe the distribution of gambling-related risk levels in the sample.

The descriptive results for participation frequency and time spent per gambling episode provided the basis for the subsequent Latent Profile Analysis (LPA). These two indicators were used to identify distinct patterns of participation in online gambling activities, whereas MAGS scores were analyzed separately to describe gambling-related risk and were not included in the estimation of the latent profiles.

To address the second objective, Latent Profile Analysis (LPA) was conducted using Mplus to identify distinct patterns of online gambling participation based on two indicators: participation frequency (episodes per week) and time spent per gambling episode (minutes). Models specifying two, three, four, and five profiles were estimated and compared. Model selection was based on the Akaike Information Criterion (AIC), Bayesian Information Criterion (BIC), adjusted Bayesian Information Criterion (aBIC), entropy, the Lo–Mendell–Rubin adjusted likelihood ratio test (LMR-LRT), and the bootstrap likelihood ratio test (BLRT). Profile sizes, model convergence and stability, classification quality, and substantive interpretability were also considered. Lower AIC, BIC, and aBIC values indicated better relative model fit, while entropy was used to assess classification accuracy. The likelihood ratio tests were used to evaluate whether adding a profile significantly improved model fit. The final solution was selected by considering these statistical criteria together with profile size and interpretability.

After selecting the final model, the number and percentage of participants assigned to each profile were calculated, along with the mean participation frequency and mean duration per episode. Profiles were labelled according to their relative levels of participation frequency and duration.

Gambling-related risk, assessed using the total Massachusetts Gambling Screen (MAGS) score, was treated as an external variable and was not included in the estimation of the latent profiles. Differences in MAGS scores across profiles were examined using the Bolck–Croon–Hagenaars (BCH) procedure in Mplus, which accounts for uncertainty in latent profile classification when comparing an external outcome across profiles. An overall Wald chi-square test was used to assess differences in MAGS scores across profiles, followed by pairwise comparisons. Adjusted *p*-values were used to evaluate pairwise differences, with statistical significance set at *p* < 0.05.

Finally, the association between latent profile membership and gambling modality was examined using Pearson’s chi-square test of independence to determine whether the distribution of gambling modalities differed across profiles. Expected cell frequencies were examined to assess the assumptions of the test. Where these assumptions were not met, an appropriate alternative procedure was considered. Statistical significance was set at *p* < 0.05.

To address the third objective, differences in anxiety and depression across the three latent profiles of online gambling and betting participation were examined using the Bolck–Croon–Hagenaars (BCH) procedure in Mplus. This method accounts for uncertainty in latent profile classification when comparing distal outcomes across profiles, thereby reducing potential bias associated with conventional analyses based on assigning participants to their most likely profile.

Anxiety and depression scores were treated as distal outcome variables and were not included in the estimation of the latent profiles. For each outcome, an overall Wald chi-square test was used to assess whether mean scores differed significantly across the three profiles. When the overall test was statistically significant, pairwise comparisons were conducted to identify differences between specific profiles. Adjusted *p*-values were used to account for multiple comparisons. Descriptive statistics were also examined to characterize the distribution of anxiety and depression scores across profiles and to determine the direction of the observed differences. Statistical significance was set at *p* < 0.05.

To address the fourth objective, Pearson’s product–moment correlation analyses were conducted to examine the associations between online gambling risk and the emotional variables of anxiety and depression. Online gambling risk was assessed using the total score on the Massachusetts Gambling Screen (MAGS), while anxiety and depression were assessed using their respective scale scores.

The Pearson correlation coefficient (r) was calculated to determine the direction and strength of the linear associations between MAGS scores, anxiety, and depression. Specifically, correlations were examined between MAGS scores and depression, MAGS scores and anxiety, and anxiety and depression. All variables were treated as continuous measures, and statistical significance was assessed using two-tailed tests, with the significance level set at *p* < 0.05.

MAGS scores were treated as an external variable and were not included in the estimation of the latent participation profiles. The analyses were intended to examine associations between gambling-related risk and emotional problems rather than to establish causal relationships. Given the cross-sectional and observational design of the study, the findings were interpreted as associations and not as evidence of causality.

## 3. Results

*Objective 1. Frequency of Participation, Time Spent per Online Gambling Episode, and Gambling-Related Risk*.

As shown in Table 1, of the 318 participants, 95 (29.9%) reported participating in online gambling approximately once a month, while 91 (28.6%) reported participating several times a month. Additionally, 83 participants (26.1%) reported participating several times a week, and 49 (15.4%) reported participating every day. Overall, these findings indicate considerable variability in participation frequency, with 41.5% of participants reporting participation in online gambling several times a week or every day.

Regarding time spent per gambling episode, 109 participants (34.3%) reported spending less than 20 min per episode, 92 (28.9%) between 20 and 39 min, 68 (21.4%) between 40 and 59 min, and 49 (15.4%) 60 min or more. Thus, although the largest proportion of participants reported episodes lasting less than 20 min, 36.8% spent at least 40 min per episode.

Gambling-related risk was assessed separately using the Massachusetts Gambling Screen (MAGS). Based on the revised cut-off criteria, participants were classified into three categories: low or no risk (MAGS score < 0), moderate risk (MAGS score ≥ 0 and <2), and high risk or probable pathological gambling (MAGS score ≥ 2). Of the 318 participants, 157 (49.4%) were classified as having low or no gambling-related risk, 86 (27.0%) as having moderate risk, and 75 (23.6%) as having high risk or probable pathological gambling (Table 2).

The descriptive results for participation frequency and time spent per gambling episode provided the basis for the subsequent Latent Profile Analysis (LPA), which aimed to identify distinct patterns of online gambling participation among young people with reduced mobility. These two indicators were used to construct the latent profiles, whereas MAGS scores were used separately to assess gambling-related risk and were not included in the profile estimation.

*Objective 2. To explore the potential existence of distinct profiles of online gambling participation intensity based on participation frequency and time spent per gambling episode.*

Latent Profile Analysis (LPA) was conducted to identify distinct patterns of online gambling participation based on participation frequency and duration per episode. Two-, three-, four-, and five-profile solutions were estimated and compared using the Akaike Information Criterion (AIC), Bayesian Information Criterion (BIC), adjusted BIC (aBIC), entropy, profile sizes, model convergence and stability, and substantive interpretability. The Lo–Mendell–Rubin adjusted likelihood ratio test (LMR-LRT) and bootstrap likelihood ratio test (BLRT) were also considered.

As shown in Table 3, the three-profile solution yielded the lowest AIC (4138.20), BIC (4190.87), and aBIC (4146.46) values among the models examined, together with an entropy value of 0.86, indicating good classification accuracy. The LMR-LRT indicated a significant improvement when moving from the two-profile to the three-profile solution (*p* = 0.012), whereas adding a fourth (*p* = 0.180) or fifth profile (*p* = 0.420) did not significantly improve the solution according to this test. Although the BLRT remained significant across the model comparisons (*p* = 0.001), the four- and five-profile solutions included relatively small groups, representing 7.5% and 4.1% of the sample, respectively. Considering the combined evidence from the information criteria, likelihood ratio tests, classification quality, profile sizes, and substantive interpretability, the three-profile solution was retained as the most parsimonious and interpretable representation of online gambling participation patterns.

The retained three-profile solution comprised 318 participants distributed across three distinct patterns of online gambling participation. Profile 1 included 150 participants (47.2%) and showed the lowest participation frequency (0.8 episodes per week) and the shortest mean duration (17.6 min per episode). This profile was labelled *Infrequent and non-regular participation*.

Profile 2 comprised 125 participants (39.3%), with a mean frequency of 2.4 episodes per week and a mean duration of 35.8 min per episode. It was labelled *Frequent and moderate participation*, reflecting an intermediate level of participation in comparison with the other profiles. Profile 3 included 43 participants (13.5%) and showed the highest mean frequency (5.1 episodes per week) and the longest mean duration (68.7 min per episode). Compared with Profile 2, participants in Profile 3 engaged in online gambling more than twice as frequently and spent almost twice as long per episode, indicating a substantially more intensive participation pattern. Accordingly, this profile was labelled *Frequent and intensive participation*.

Descriptive statistics also suggested a gradual increase in gambling-related risk across the three latent profiles. As shown in Table 4, the mean MAGS score was 1.20 (SD = 0.80) in Profile 1, 2.60 (SD = 1.10) in Profile 2, and 5.10 (SD = 1.40) in Profile 3. To examine differences in gambling-related risk across profiles while accounting for uncertainty in latent profile classification, the Bolck–Croon–Hagenaars (BCH) procedure was used.

The overall BCH Wald test indicated statistically significant differences in MAGS scores across the three profiles (Wald χ^2^(2) = 18.60, *p* < 0.001). Pairwise comparisons showed significant differences between Profile 1 and Profile 2 (adjusted *p* = 0.012), Profile 1 and Profile 3 (adjusted *p* = 0.001), and Profile 2 and Profile 3 (adjusted *p* = 0.018). The pattern of results indicated progressively higher gambling-related risk across the profiles, with the highest scores observed among participants characterized by the most frequent and intensive online gambling participation.

Once the profiles had been identified, their associations with other study variables were examined. Gambling risk, sex, age, and gambling modality were not included as indicators in the LPA model but were considered subsequently to characterize the profiles and explore their relationships with other variables.

Regarding gambling modality, a statistically significant association with profile membership was observed, χ^2^(8) = 24.83, *p* = 0.002. Participants belonging to the *Frequent and intensive participation* profile were more highly represented in sports betting and online casino modalities, whereas participants who did not engage in online gambling were concentrated primarily in the *Infrequent and non-regular participation* profile.

*Objective 3. Differences in Anxiety and Depression According to Latent Participation Profiles.*

The differences in psychological distress across the three latent profiles were examined using the Bolck–Croon–Hagenaars (BCH) procedure. As shown in Table 5, the overall BCH test indicated statistically significant differences in depression across profiles, χ^2^(2) = 12.64, *p* = 0.002. Mean depression scores increased across the profiles, from 1.45 (*SD* = 0.62) in the Occasional and Brief profile to 1.82 (*SD* = 0.68) in the Frequent and Moderate profile and 2.31 (*SD* = 0.74) in the Frequent and Intensive profile.

A similar pattern was observed for anxiety. The overall BCH test indicated statistically significant differences between profiles, χ^2^(2) = 10.83, *p* = 0.004. Mean anxiety scores were 1.38 (*SD* = 0.59) in the Occasional and Brief profile, 1.76 (*SD* = 0.65) in the Frequent and Moderate profile, and 2.20 (*SD* = 0.72) in the Frequent and Intensive profile (Table 5).

*Objective 4. Association Between Online Gambling Risk and Emotional Problems.*

**Figure 1:**
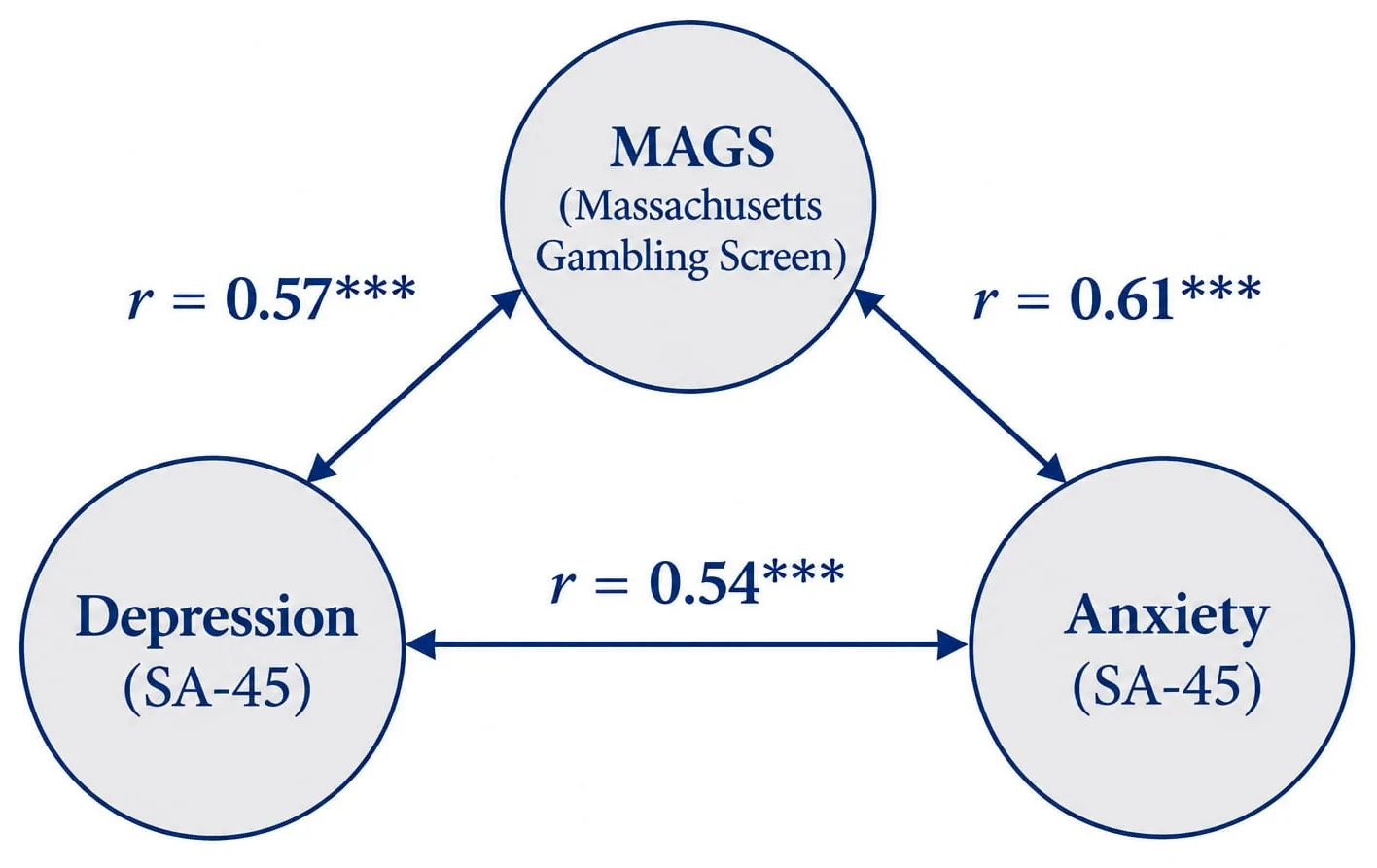
Pearson Correlations Between Online Gambling Risk, Anxiety, and Depression. Note. MAGS = Massachusetts Gambling Screen; SA-45 = Symptom Assessment-45. Correlation coefficients are Pearson’s r. *** *p* < 0.001. All associations are positive and statistically significant.

Once the latent participation profiles had been identified based on participation frequency and time spent per gambling episode, the associations between online gambling risk and the emotional variables of anxiety and depression were examined. Gambling risk was treated as an external variable and was not included in the construction of the latent profiles (Figure 1).

Pearson correlation analyses were conducted to examine the associations between online gambling risk, assessed using the total score on the Massachusetts Gambling Screen (MAGS), and depression and anxiety scores. The results indicated positive associations between gambling risk and both emotional dimensions. Specifically, higher MAGS scores were associated with higher depression scores (*r* = 0.57, *p* < 0.001) and higher anxiety scores (*r* = 0.61, *p* < 0.001). Anxiety and depression were also positively correlated (*r* = 0.54, *p* < 0.001). These findings indicate that greater online gambling risk was associated with higher levels of anxiety and depression. However, given the cross-sectional and observational nature of the data, these associations should not be interpreted as evidence of causal relationships.

## 4. Discussion

The findings of this study indicate that participation in online gambling, including online games of chance and sports betting, among young people with reduced mobility is heterogeneous. The analysis identified three distinct profiles based on participation frequency and time spent per episode: Profile 1, Infrequent and non-regular participation; Profile 2, Frequent and Moderate Participation; and Profile 3, Frequent and Intensive Participation. The BCH analyses revealed statistically significant differences in anxiety and depression across the three profiles. In addition, the structural equation model showed positive associations between online gambling risk and both emotional outcomes. Overall, these findings extend the existing evidence regarding the heterogeneity of online gambling participation patterns and their association with indicators of psychological vulnerability.

Regarding the first objective, the results showed considerable variability in the frequency of and time spent participating in online gambling activities, including online games of chance and sports betting. This heterogeneity is particularly relevant in digital environments, which are characterized by high accessibility, availability, and diversity of gambling modalities [3,5]. The systematic review by Ghelfi et al. [5] specifically highlights these characteristics as relevant factors for understanding risk and protective factors associated with online gambling.

The literature specifically addressing disability also supports the relevance of examining disability and gambling jointly. Morasco and Petry [9] found a higher prevalence of disordered gambling among people receiving disability benefits, as well as an association with poorer physical and mental functioning. Similarly, Jacob et al. [8], using a representative UK sample, reported an association between problem gambling and functional disability, while Kolberg et al. [10] provided longitudinal evidence concerning the relationship between disability benefits and gambling disorder. These findings reinforce the importance of examining gambling behavior in populations with disabilities, although the samples and study designs differ from those of the present study.

In this regard, our findings move beyond a general comparison between people with and without disabilities by examining variability within a population of young people with reduced mobility. Nevertheless, higher participation frequency or longer time spent per gambling episode does not, in itself, imply the presence of problem gambling. These variables are indicators of participation intensity and should be interpreted in conjunction with gambling risk, behavioral consequences, and psychological well-being.

With regard to the second objective, the latent profile analysis identified three distinct participation patterns: Profile 1, Infrequent and non-regular participation; Profile 2, Frequent and moderate participation; and Profile 3, Frequent and intensive participation. This finding supports the existence of different configurations of online gambling participation and goes beyond an exclusively dichotomous approach based on whether individuals participate or do not participate in online gambling activities.

The observed heterogeneity is consistent with the *Pathways Model* proposed by Blaszczynski and Nower [15], which describes different pathways toward problem gambling. Subsequently, Nower et al. [18] revised this model while retaining the conceptualization of distinct gambler subtypes. Similarly, Kurilla [17], in a systematic review, found support for the existence of three subtypes among adults, although greater heterogeneity of findings was reported among adolescents. Black and Allen [16], using latent class analysis in a non-clinical sample, also provided evidence of different gambler subtypes.

However, the profiles identified in the present study should not be considered equivalent to the subtypes proposed by the *Pathways Model*. Whereas this model incorporates etiological, psychological, and motivational variables, our profiles were derived exclusively from participation frequency and time spent per gambling episode. Therefore, they represent empirical configurations of participation intensity rather than diagnostic categories or etiological trajectories.

This distinction also provides context for the findings reported by Ayala-Rojas et al. [6], who identified differences between individuals seeking treatment for online gambling disorder and those seeking treatment for sports betting. Although their population and methodology differ from those of the present study, their findings support the existence of distinct characteristics associated with different forms of gambling participation.

Regarding the third objective, the BCH procedure identified statistically significant differences in anxiety and depression across the three latent profiles, according to the overall Wald tests. The pairwise BCH comparisons provided further information about differences between specific profiles. These findings are consistent with previous research linking greater involvement in problematic gambling with anxiety and depressive symptoms. Nower et al. [18] identified a subtype characterized by greater emotional vulnerability and higher levels of anxiety and depression. Similarly, Jauregui et al. [23] reported associations between pathological gambling, difficulties in emotion regulation, and anxiety and depressive symptoms. Yakovenko and Hodgins [25] also highlighted the substantial co-occurrence of gambling-related problems and different mental health problems.

These findings are particularly relevant among young people with disabilities. Gorter et al. [27] emphasized the importance of psychological and contextual factors related to mental health among adolescents and young adults with cerebral palsy, while Bhatnagar et al. [26] reported higher rates of certain mental health diagnoses among children and young people with cerebral palsy. Rausch et al. [28], in a recent review, likewise highlighted the relevance of mental health problems in this population.

Accordingly, our findings suggest that different patterns of online gambling participation are associated with differences in anxiety and depression. However, given the cross-sectional nature of the study, the direction of this association cannot be determined.

Anxiety and depression may contribute to greater involvement in gambling, intensive participation may be associated with greater psychological distress, or both may be influenced by other factors.

Regarding the fourth objective, the Pearson correlation analyses revealed positive and statistically significant associations between online gambling risk and both depression and anxiety. Specifically, higher MAGS scores were associated with higher depression and anxiety scores. Anxiety and depression were also positively correlated. These findings indicate that online gambling risk and emotional distress are related in young people with reduced mobility, although the strength of these associations varied across the variables examined.

These findings are consistent with previous evidence concerning the relationship between gambling-related problems and psychological distress. Suriá-Martínez et al. [14], in a Spanish university population, observed differences in psychopathological symptoms associated with online gambling risk profiles.

Similarly, Morasco and Petry [9] found an association between disordered gambling and poorer physical and mental functioning among people with disabilities, while Jacob et al. [8] reported an association between problem gambling and functional disability.

The relationship between online gambling risk and emotional distress can also be discussed in relation to emotion regulation. Velotti et al. [22], in a systematic review and meta-analysis, found an association between gambling disorder and difficulties in emotion regulation, while Jauregui et al. [23] identified relationships between emotion regulation and anxiety and depressive symptoms. Although these findings provide a relevant theoretical context for interpreting the correlations observed in the present study, emotion regulation was not directly assessed and therefore cannot be evaluated as an explanatory factor in the current sample.

The characteristics of the online environment also provide relevant context for understanding patterns of gambling participation. Ghelfi et al. [5] highlight accessibility, availability, anonymity, and the diversity of gambling modalities as specific characteristics of online gambling. Using behavioral data, Ukhov et al. [7] also showed that different forms of online gambling may display distinct behavioral patterns, while Ayala-Rojas et al. [6] identified differences between online gambling and sports betting. These studies underscore the heterogeneity of online gambling activities and the importance of distinguishing participation patterns from gambling-related risk.

Among people with disabilities, Pitt et al. [3] noted that gambling-related environments may simultaneously serve recreational and social functions while also presenting potential risks. Pitt et al. [4] similarly showed, among people with intellectual disabilities, the importance of understanding different forms of participation and pathways to gambling initiation and maintenance. Although these populations are not equivalent to the population with reduced mobility examined in the present study, their findings support the need to consider disability as a specific contextual factor.

This issue has gained further relevance through recent work such as that of Jang et al. [11], who highlighted the need for greater attention to the relationship between disability and gambling-related harms. The present findings contribute to this line of research by examining participation intensity, online gambling risk, anxiety, and depression within a sample of young people with reduced mobility. In particular, the results distinguish between participation patterns, assessed through frequency and time spent per gambling episode, and gambling risk, assessed using the MAGS. This distinction is important because participation intensity and gambling-related risk represent different dimensions of gambling behavior.

Three complementary theoretical perspectives provide a framework for contextualizing these findings. The *Pathways Model* [15,16,17,18] provides a framework for contextualizing the heterogeneity of gambling behavior, although our profiles do not constitute a replication of its subtypes. The I-PACE model [19,20] provides a framework for understanding the interaction between individual characteristics, affective states, cognitive processes, and addictive behaviors. Finally, the emotion regulation perspective [22,23] offers a potential theoretical explanation for the coexistence of higher gambling risk and psychological distress. These frameworks offer complementary interpretations of the findings; however, the present study did not directly test the mechanisms proposed by these models.

Overall, the Pearson correlations indicate that higher online gambling risk scores were associated with greater anxiety and depression among young people with reduced mobility. These findings highlight the relevance of considering gambling-related risk and emotional well-being together in this population. Nevertheless, the results should be interpreted within the scope of the variables assessed and the study design, and further research is needed to examine these associations in more diverse samples and across different patterns of online gambling participation.

From a prevention perspective, the findings suggest that assessment should not be limited to determining whether an individual participates in online gambling, but should also consider participation frequency, time spent gambling online, gambling risk, and indicators of psychological well-being. Profile 3, Frequent and Intensive Participation, may warrant closer preventive assessment because it represents the most intensive participation pattern. However, the interpretation of this profile in relation to anxiety and depression should be based on the specific pairwise BCH results. Membership in this profile should not be interpreted as equivalent to gambling disorder. Similarly, Profile 1, Infrequent and Brief Participation, describes a pattern of lower participation intensity, but should not automatically be interpreted as indicating an absence of gambling-related risk.

As with any empirical study, several limitations and directions for future research should be considered. First, the cross-sectional design represents the main limitation, as it prevents the establishment of temporal or causal relationships among participation, gambling risk, anxiety, and depression. Second, the use of self-report measures may introduce recall and response biases. Third, potential confounding variables, including age, sex, degree of disability, and mobility status, were not controlled for in the analyses. These factors may influence participation patterns, gambling-related risk, and emotional well-being, potentially affecting the observed associations. Therefore, the findings should be interpreted with caution, and the possible influence of these variables cannot be ruled out. Finally, the identified profiles represent statistical configurations derived from the variables included in the analysis and should not be regarded as stable clinical categories.

Future research should employ longitudinal designs to examine the stability of these profiles and potential transitions between participation patterns. Further studies should also incorporate adjusted analyses that explicitly account for age, sex, degree of disability, and mobility status to determine whether the observed associations between participation profiles, gambling-related risk, anxiety, and depression remain after controlling for these potential confounders. This would help clarify the contribution of these factors and provide a more comprehensive understanding of the relationships under investigation. In addition, future studies should incorporate variables related to emotion regulation, coping, loneliness, social support, impulsivity, cognitive distortions, gambling motives, and perceived consequences. Further research should also evaluate the effectiveness of prevention and rehabilitation programs tailored to the needs of young people with reduced mobility, considering the different participation profiles identified in this study. Such programs could incorporate early screening for gambling-related risk, psychological support, emotional regulation and coping strategies, and accessible interventions adapted to the specific needs and circumstances of this population. Evaluating these approaches would help determine which strategies are most effective in reducing gambling-related harm and promoting psychological well-being. The longitudinal work of Kolberg et al. [10] illustrates the value of such approaches for examining the temporal relationship between disability and gambling-related problems.

## 5. Conclusions

Overall, the findings indicate that online gambling participation among young people with reduced mobility can be characterized by three distinct participation patterns: infrequent and non-regular participation, frequent and moderate participation, and frequent and intensive participation. The BCH analyses revealed statistically significant differences in anxiety and depression across the three profiles, while online gambling risk was positively associated with both emotional outcomes.

These findings extend previous research on the heterogeneity of gambling participation [15,16,17,18] and provide specific evidence concerning young people with reduced mobility, a population that remains relatively understudied [8,9,10,11]. They also contribute to the understanding of the associations between online gambling participation, gambling-related risk, and psychological distress [5,22,23,25].

Nevertheless, the findings should be interpreted as associations and empirical participation configurations rather than as evidence of causality or clinical categories. This distinction is particularly important when informing future longitudinal research and preventive strategies for young people with reduced mobility.

## Figures and Tables

**Table 1:** Participation frequency and time spent per gambling episode.

Variable	Category	n	%
Participation frequency	Approximately once a month	95	29.9
	Several times a month	91	28.6
	Several times a week	83	26.1
	Every day	49	15.4
Time per participation episode	Less than 20 min	109	34.3
	20–39 min	92	28.9
	40–59 min	68	21.4
	≥60 min	49	15.4
Total		318	100.0

**Table 2:** Distribution of participants by gambling-related risk level (MAGS).

Gambling-Related Risk Level	MAGS Score	n	%
Low or no risk	Score < 0	157	49.4
Moderate risk	0 ≤ Score < 2	86	27.0
High risk or probable pathological gambling	Score ≥ 2	75	23.6
Total	—	318	100.0

**Table 3:** Comparison of latent profile models.

Profile	Log-Likelihood (LL)	Free Parameters (k)	AIC	BIC	aBIC	Entropy	LMR-LRT (p)	BLRT (p)	Smallest Profile (%)
2 profiles	−2100.18	9	4218.36	4252.22	4223.67	0.79	0.001	0.001	35.2
*3 profiles*	*−2055.10*	*14*	*4138.20*	*4190.87*	*4146.46*	*0.86*	*0.012*	*0.001*	*13.5*
4 profiles	−2052.42	19	4142.84	4214.32	4154.06	0.82	0.180	0.001	7.5
5 profiles	−2051.58	24	4151.16	4241.45	4165.33	0.79	0.420	0.001	4.1

*Note.* N = 318. LL = log-likelihood; *k* = number of free parameters; AIC = Akaike Information Criterion; BIC = Bayesian Information Criterion; aBIC = adjusted Bayesian Information Criterion; LMR-LRT = Lo-Mendell-Rubin likelihood ratio test; BLRT = bootstrap likelihood ratio test.

**Table 4:** Characterization of latent profiles according to participation indicators.

Latent Profile	Mean Frequency (Episodes/Week)	Mean Time (Minutes)	n	%	Gambling-Risk Score, M (SD)
Profile 1. *Infrequent and non-regular participation*	0.8	17.6	150	47.2	1.20 (0.80)
Profile 2. *Frequent and moderate participation*	2.4	35.8	125	39.3	2.60 (1.10)
Profile 3. *Frequent and intensive participation*	5.1	68.7	43	13.5	5.10 (1.40)
Total	—	—	318	100.0	2.28 (1.64)

**Table 5:** Anxiety and depression across latent online gambling and betting participation profiles.

Variable	Profile 1: Occasional and Brief (*n* = 150)	Profile 2: Frequent and Moderate (*n* = 125)	Profile 3: Frequent and Intensive (*n* = 43)	Overall BCH Test	Pairwise Comparisons
Depression *M* (*SD*)	1.45 (0.62)	1.82 (0.68)	2.31 (0.74)	χ^2^(2) = 12.64, *p* = 0.002	P1 < P2; P1 < P3; P2 < P3
Anxiety *M* (*SD*)	1.38 (0.59)	1.76 (0.65)	2.20 (0.72)	χ^2^(2) = 10.83, *p* = 0.004	P1 < P2; P1 < P3; P2 < P3

*Note.* Values are expressed as means (M) and standard deviations (SD). Wald χ^2^ represents the Wald chi-square statistic from the BCH procedure. Pairwise comparisons are based on the BCH procedure; the *p*-values must be verified against the original Mplus output, including confirmation of the multiple-comparison adjustment used. P1 = Profile 1, Infrequent and non-regular participation; P2 = Profile 2, Frequent and moderate participation; P3 = Profile 3, Frequent and intensive participation.

## Data Availability

The data are not available because the database used contains sensitive information related to students, including data associated with disability conditions.

## References

[1] Organización Mundial de la Salud (2023). Discapacidad. https://www.who.int/es/news-room/fact-sheets/detail/disability-and-health.

[2] Poon E.C.S., Soh X.C., Poh T.J.H., Tan A.C.S., Hartanto A. (2026). Global Prevalence of Internet Gaming Disorder: An Umbrella Review of Meta-Analytic Evidence and Implications for Child and Adolescent Psychiatry. Brain Sci..

[3] Pitt H., Thomas S.L., Watson J., Shuttleworth R., Murfitt K., Balandin S. (2020). Weighing up the risks and benefits of community gambling venues as recreational spaces for people with lifelong disability. BMC Public Health.

[4] Pitt H., Thomas S.L., Watson J., Shuttleworth R., Murfitt K., Balandin S. (2021). How do people with intellectual disability engage with and understand gambling? A qualitative study of adults in Victoria, Australia. Front. Public Health.

[5] Ghelfi M., Scattola P., Giudici G., Velasco V. (2024). Online gambling: A systematic review of risk and protective factors in the adult population. J. Gambl. Stud..

[6] Ayala-Rojas R.E., Granero R., Fernández-Aranda F., Gómez-Peña M., Moragas L., Perales I., Jiménez-Murcia S. (2025). Online gaming disorder and sports betting addiction: Convergences and divergences. Int. J. Ment. Health Addict..

[7] Ukhov I., Bjurgert J., Auer M., Griffiths M.D. (2021). Online problem gambling: A comparison of casino players and sports bettors via predictive modeling using behavioral tracking data. J. Gambl. Stud..

[8] Jacob L., Lopez-Sanchez G.F., Oh H., Kostev K., Koyanagi A., Haro J.M., Pardhan S., Shin J.I., Smith L. (2022). Association between problem gambling and functional disability: A nationally representative study conducted in the United Kingdom. J. Addict. Med..

[9] Morasco B.J., Petry N.M. (2006). Gambling problems and health functioning in individuals receiving disability. Disabil. Rehabil..

[10] Kolberg E., Smith O.R.F., Leino T., Pallesen S., Griffiths M.D., Mentzoni R.A., Syvertsen A. (2025). Disability benefit and gambling disorder: A longitudinal study based on national registry data. J. Gambl. Stud..

[11] Jang S., Ju H.J., Whelan J.P. (2026). Gambling and disability: An overlooked context for gambling harm. J. Gambl. Stud..

[12] Suriá-Martínez R., García-Castillo F., López-Sánchez C., Villegas-Castrillo E., Carretón-Ballester C. (2024). Online games and cognitive distortions: A comparative analysis in students with and without disabilities. Eur. J. Investig. Health Psychol. Educ..

[13] Suriá-Martínez R., García-Castillo F., Villegas-Castrillo E., López-Sánchez C., Carretón-Ballester C. (2024). Negative impact of online gambling problematic in disabled and non-disabled university students: Exploring the risk profile. Front. Psychol..

[14] Suriá-Martínez R., García-Castillo F., López-Sánchez C., García del Castillo J.A. (2025). Sintomatología psicopatología en estudiantes universitarios con y sin discapacidad participantes de juegos y apuestas online. Retos.

[15] Blaszczynski A., Nower L. (2002). A pathways model of problem and pathological gambling. Addiction.

[16] Black D.W., Allen J. (2022). Testing the validity of the Pathways Model: A latent class analysis of potential pathological gambling subtypes in a non-treatment sample. J. Gambl. Stud..

[17] Kurilla A. (2021). Is subtyping of gamblers based on the Pathways Model of problem and disordered gambling valid? A systematic review. J. Gambl. Stud..

[18] Nower L., Blaszczynski A., Li W.A. (2022). Clarifying gambling subtypes: The revised pathways model of problem gambling. Addiction.

[19] Brand M., Young K.S., Laier C., Wölfling K., Potenza M.N. (2016). Integrating psychological and neurobiological considerations regarding the development and maintenance of specific Internet-use disorders: An Interaction of Person-Affect-Cognition-Execution (I-PACE) model. Neurosci. Biobehav. Rev..

[20] Brand M., Wegmann E., Stark R., Müller A., Wölfling K., Robbins T.W., Potenza M.N. (2019). The Interaction of Person-Affect-Cognition-Execution (I-PACE) model for addictive behaviors: Update, generalization to addictive behaviors beyond Internet-use disorders, and specification of the process character of addictive behaviors. Neurosci. Biobehav. Rev..

[21] Rogier G., Velotti P. (2018). Conceptualizing gambling disorder with the process model of emotion regulation. J. Behav. Addict..

[22] Velotti P., Rogier G., Beomonte Zobel S., Billieux J. (2021). Association between gambling disorder and emotion (dys)regulation: A systematic review and meta-analysis. Clin. Psychol. Rev..

[23] Jauregui P., Estévez A., Urbiola I. (2016). Pathological gambling and associated drug and alcohol abuse, emotion regulation, and anxious-depressive symptomatology. J. Behav. Addict..

[24] Jauregui P., Urbiola I., Estévez A. (2016). Metacognition in pathological gambling and its relationship with anxious and depressive symptomatology. J. Gambl. Stud..

[25] Yakovenko I., Hodgins D.C. (2017). A scoping review of co-morbidity in individuals with disordered gambling. Int. Gambl. Stud..

[26] Bhatnagar S., Mitelpunkt A., Rizzo J.J., Zhang N., Guzman T., Schuetter R., Vargus-Adams J., Bailes A.F., Greve K., Gerstle M. (2024). Mental health diagnoses risk among children and young adults with cerebral palsy, chronic conditions, or typical development. JAMA Netw. Open.

[27] Gorter J.W., Fehlings D., Ferro M.A., Gonzalez A., Green A.D., Hopmans S.N., McCauley D., Palisano R.J., Rosenbaum P., Speller B. (2022). Correlates of mental health in adolescents and young adults with cerebral palsy: A cross-sectional analysis of the MyStory Project. J. Clin. Med..

[28] Rausch R., Chahin S., Miller C., Dopheide L., Bovio N., Harris A., Patel D. (2025). The mental health of children with cerebral palsy: A review of the last five years of research. J. Clin. Med..

[29] Li J., Li Y., Mei X., He H., Yu L., Ho G.W.K., Chan E.A. (2024). Effectiveness of cognitive-based interventions on psychological distress in adolescents with physical disabilities: A systematic review and meta-analysis. Int. J. Ment. Health Nurs..

[30] Shaffer H.J., LaBrie R., Scanlan K.M., Cummings T.N. (1994). Pathological gambling among adolescents: Massachusetts Gambling Screen (MAGS). J. Gambl. Stud..

[31] Secades R., Villa A. (1998). El Juego Patológico: Prevención, Evaluación y Tratamiento en la Adolescencia.

[32] Davison M.L., Bershadsky B., Bieber J., Silversmith D., Maruish M.E., Kane R.L. (1997). Development of a brief, multidimensional, self-report instrument for treatment outcomes assessment in psychiatric settings: Preliminary findings. Assessment.

[33] Sandín B., Valiente R.M., Chorot P., Santed M.A., Lostao L. (2008). SA-45: Forma abreviada del SCL-90. Psicothema.

